# DDBJ progress report: a new submission system for leading to a correct annotation

**DOI:** 10.1093/nar/gkt1066

**Published:** 2013-11-03

**Authors:** Takehide Kosuge, Jun Mashima, Yuichi Kodama, Takatomo Fujisawa, Eli Kaminuma, Osamu Ogasawara, Kousaku Okubo, Toshihisa Takagi, Yasukazu Nakamura

**Affiliations:** ^1^DDBJ Center, National Institute of Genetics, Yata 1111, Mishima, Shizuoka 411-8540, Japan and ^2^National Bioscience Database Center, Japan Science and Technology Agency, Tokyo 102-8666, Japan

## Abstract

The DNA Data Bank of Japan (DDBJ; http://www.ddbj.nig.ac.jp) maintains and provides archival, retrieval and analytical resources for biological information. This database content is shared with the US National Center for Biotechnology Information (NCBI) and the European Bioinformatics Institute (EBI) within the framework of the International Nucleotide Sequence Database Collaboration (INSDC). DDBJ launched a new nucleotide sequence submission system for receiving traditional nucleotide sequence. We expect that the new submission system will be useful for many submitters to input accurate annotation and reduce the time needed for data input. In addition, DDBJ has started a new service, the Japanese Genotype–phenotype Archive (JGA), with our partner institute, the National Bioscience Database Center (NBDC). JGA permanently archives and shares all types of individual human genetic and phenotypic data. We also introduce improvements in the DDBJ services and databases made during the past year.

## INTRODUCTION

The DNA Data Bank of Japan (DDBJ, http://www.ddbj.nig.ac.jp) (1) is one of the three databanks that comprise the DDBJ/ENA/GenBank International Nucleotide Sequence Database (INSD) (2–4), which is a close collaboration between DDBJ, the European Bioinformatics Institute (EBI) in Europe and the National Center for Biotechnology Information (NCBI) in the USA. Like ENA and GenBank, DDBJ provides biological databases and analytical services to researchers to support biological research.

We have already reported that our previous supercomputer system has been totally replaced by a new commodity-cluster-based system (1). Our services including Web services, submission system, BLAST, CLUSTALW, WebAPI and databases are now running on a new supercomputer system. In addition to a traditional assembled sequence archive, DDBJ provides the DDBJ Sequence Read Archive (DRA) (5) and the DDBJ BioProject (6) for receiving short reads from next-generation sequencing (NGS) machines and organizing the corresponding data obtained from research projects. In 2013, DDBJ has started the BioSample database in collaboration with INSDC (7,8) to organize sample information with the DRA and the BioProject. In recent years, DDBJ has devoted energy for constructing databases such as DRA and BioProject to prepare for receiving huge numbers of sequences from next-generation sequencers and organize genome projects. Because of an increase in databases in a short period of time, DDBJ has been concerned that it is becoming difficult for submitters to submit nucleotide sequences with accurate information. Although the amount of traditional assembled sequence data is smaller than that of submissions to the DRA (raw data from NGS), the annotations describing each nucleotide sequences are used for a reference database that helps researchers in genome analysis. DDBJ is aware of the importance of allowing the submission system to lead submitters to provide accurate annotation. DDBJ had long (since 1995) used SAKURA (9) to receive traditional nucleotide sequences via the Web and has replaced it with a new system operating on a new supercomputer. The new DDBJ nucleotide sequence submission system incorporates ideas that will reduce the time needed for completing a submission and help submitters provide accurate annotation.

DDBJ decided to launch Japanese Genotype–phenotype Archive (JGA) in collaboration with the National Bioscience Database Center (NBDC) of the Japan Science and Technology Agency. JGA is constructed for the purpose of archiving human personal genomic and phenotypic data and providing researchers with secure access.

All resources described here are available from http://www.ddbj.nig.ac.jp.

## DDBJ ARCHIVAL DATABASES IN 2013

### DDBJ traditional assembled sequence archive

Between July 2012 and June 2013, the DDBJ periodical release increased by 11 799 452 entries and 11 686 547 887 base pairs. This periodical release, also known as the INSD core traditional nucleotide flat files, does not include whole-genome shotgun (WGS) and third party data (TPA) files (10). The DDBJ contributed 17.8% of the entries and 12.2% of the total base pairs added to the core nucleotide data of INSD. Most of the nucleotide data records provided to the DDBJ (97.83%) were submitted by Japanese researchers and the rest coming from Korea (1.26%), China (0.54%), Colombia (0.17%), Taiwan (0.04%), USA (0.04%) and other countries and regions (0.11%). DDBJ has continuously distributed sequence data in published patent applications from the Japan Patent Office (JPO, http://www.jpo.go.jp) and the Korean Intellectual Property Office (KIPO, http://www.kipo.go.kr/en). JPO transferred its data to DDBJ directly, whereas KIPO transferred its data via an arrangement with the Korean Bioinformation Center (KOBIC). A detailed statistical breakdown of the number of records is shown on the DDBJ homepage (http://www.ddbj.nig.ac.jp/breakdown_stats/prop_ent.html). In addition to the core nucleotide data, DDBJ has released a total of 5 099 547 WGS entries, 721 TPA entries, 6374 TPA-WGS entries and 1272 TPA-CON entries as of 27 August 2013.

Noteworthy large-scale data released from DDBJ are listed in Table 1. Regarding the genome of *Theileria orientalis* strain Shintoku, which was submitted by Hokkaido University, a DDBJ annotator participated in the annotation procedure. Moreover, DDBJ has released the following: coelacanth (*Latimeria chalumnae*) genome submitted by Tokyo Institute of Technology; diamondback moth (*Plutella xylostella*) draft genome submitted by the National Institute of Agrobiological Sciences; *Jatropha curcas* genome submitted by the Kazusa DNA Research Institute; mouse (*Mus musculus*) strain MSM/Ms draft genome submitted by the National Institute of Genetics (NIG); two sets of genome survey sequences (GSS) of African clawed frog (*Xenopus laevis*) submitted by the NIG; GSS of coelacanth (*L. chalumnae*) submitted by the NIG; transcriptome shotgun assemblies (TSA) of great pond snail (*Lymnaea stagnalis*) submitted by Tokushima Bunri University; TSA of a forest soil metagenome submitted by the National Institute of Advanced Industrial Science and Technology; expressed sequence tags (EST) of a species of planarians (*Dugesia japonica*) submitted by RIKEN; EST of white-tufted-ear marmoset (*Callithrix jacchus*) submitted by the NIG.

**Table 1.** List of large-scale data released by DDBJ from July 2012 to June 2013

Type	Organism	Accession number (number of entries)
Genome	*Theileria orientalis* strain Shintoku	Chromosomes: AP011946–AP011949 (4 entries)
		Apicoplast: AP011950
		Mitochondrion: AP011951
	Coelacanth (*Latimeria chalumnae*)	Scaffold CON: DF158906–DF196766 (37 861 entries)
		WGS: BAHO01000001–BAHO01475424 (475 424 entries)
	Diamondback moth (*Plutella xylostella*)	BAGR01000001–BAGR01088530 (88 530 entries)
	*Jatropha curcas*	Scaffold CON: DF145383–DF157092 (11 710 entries)
		WGS: BABX02000001–BABX02066610 (66 610 entries)
	Mouse (*Mus musculus* MSM/Ms)	WGS: BAAG010000001–BAAG011237600 (1 237 600 entries)
GSS	African clawed frog (*Xenopus laevis*)	GA131508–GA388245 (251 621 entries; some missing)
		GA720358–GA867435 (147 078 entries)
	Coelacanth (*L. chalumnae*)	GA605430–GA720357 (114 928 entries)
TSA	Great pond snail (*Lymnaea stagnalis*)	FX180119–FX296473 (116 355 entries)
	Forest soil metagenome	FX000001–FX056084 (56 084 entries)
EST	Planarian (*Dugesia japonica*)	5′-EST: FY925127–FY960824 (35 698 entries)
		3′-EST: FY960825–FY979285 (18 461 entries)
	White-tufted-ear marmoset (*Callithrix jacchus*)	HX373156–HX663542 (290 387 entries)

### Sequence read archive, BioProject and BioSample databases

As an INSDC activity, the DDBJ decided to launch the BioSample database to organize sample information across archival databases. The study and sample objects of the Sequence Read Archive will be migrated to the BioProject and BioSample records, respectively.

### Japanese Genotype–phenotype Archive

DDBJ has started a new service, the Japanese Genotype–phenotype Archive (JGA, http://trace.ddbj.nig.ac.jp/jga) with our partner institute, the NBDC. JGA is a service for permanent archiving and sharing of all types of personal genetic and phenotypic data resulting from biomedical research projects. JGA contains exclusive data collected from individuals whose consent agreements authorize data release only for specific research use. JGA provides controlled access to individual data as the database of Genotypes and Phenotypes (dbGaP) at NCBI (11) and the European Genome–phenome Archive (EGA) at EBI (12).

JGA accepts only de-identified data with a NBDC approved access plan. Users directly apply for data submission to NBDC, and JGA will accept and process submissions only when notification of a successful application process has been passed from NBDC to JGA. The accepted data types include manufacturer-specific raw data formats from array based and new sequencing platforms. The processed data, including genotype and structural variants or any summary statistical analyses, are stored in databases. JGA also accepts and distributes any phenotype data associated with the samples.

JGA implements access-granting policy, whereby decisions on granting access to the data reside with NBDC. Users must be approved by NBDC to access the JGA data.

## DDBJ SYSTEMS PROGRESS

### A new submission system: D-easy

DDBJ has launched a new Web-based nucleotide sequence submission system (code name: D-easy) for receiving traditional submissions after 3 October 2012. A former Web-based submission system, SAKURA, was terminated on 31 October 2012. SAKURA was originally created for the submission of small-scale data in 1995 and used for a long period. In recent years, we suspected that SAKURA was not suitable for the current volume of nucleotide data registration, given the large number of nucleotide sequences that can now be acquired rapidly and cheaply. Accordingly, DDBJ decided to develop a new nucleotide data submission system that works on a Web server. The new submission system features an improved sequence and annotation input system to accept larger numbers of nucleotide sequence data at once. We expect that submitters can shorten the time required for nucleotide data submission by using the new nucleotide data submission tool.

We identified problematic issues in using SAKURA, which should be improved in a new submission tool. In particular, submission of multiple nucleotide sequences was very time-consuming because nucleotide sequences were required to be submitted one by one; multiple-FASTA files were not accepted. It was difficult for a submitter to identify the feature key(s) best matching the annotation of a nucleotide sequence, leading to frequent failure of the submitter to input essential values. Sometimes we could not contact a submitter because of a mistyped e-mail address. In view of these problems of SAKURA, we identified ideas that would be helpful for inputting annotation and nucleotide sequence:

To reduce the time required for inputting annotation, a spreadsheet input method will be better.
For typical annotation, such as 16S rRNA, mRNA and D-loop, an annotation template system would be useful. Each template has feature and qualifier sets that are mandatory for each annotation. For example, when the 16S rRNA template is selected, source and rRNA feature are automatically chosen.

Finally, we equipped D-easy with the following functions.

**Figure 1.**
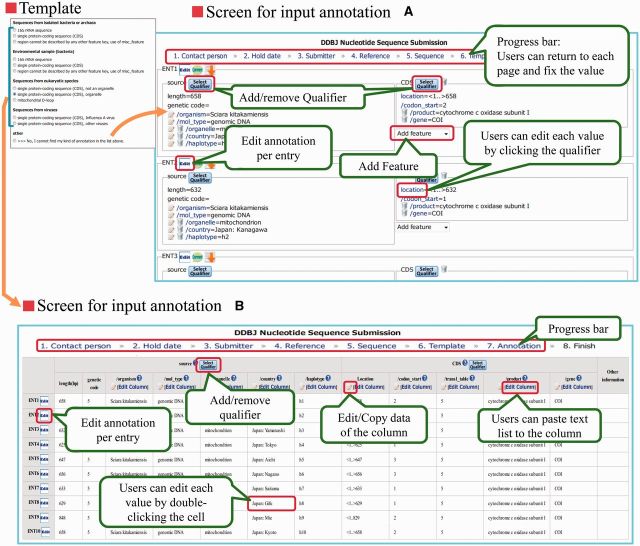
Screen shot of a new nucleotide submission system, D-easy. In general, the submitter uses a non-spreadsheet input method (**A**). When the submitter selects an annotation pattern that is prepared as a template page (e.g., 16S rRNA sequence) spreadsheet input is available (**B**).

- The e-mail address is authenticated during submission. A submitter, after inputting contact information, receives an e-mail message and must click on a link in the message to input the nucleotide data. This function obviates sending a fax to the submitter to know a correct e-mail address.
- In general, a submitter uses a non-spreadsheet input system, which is a standard annotation input form of D-easy (Figure 1A). All feature and qualifier keys, which are allowed in DDBJ's annotation rule, are selectable and submitter can input any type of annotation. Only source feature is displayed at the start screen of the input form and the submitter must add required feature keys to fill the annotation. Mandatory or recommended qualifiers are automatically chosen when a feature is added. A submitter will use the non-spreadsheet type annotation input form when ‘No, I cannot find my kind of annotation in the list above’ is chosen on the template selection page. If the annotation pattern of entire nucleotide sequences is included in the prepared template, the submitter can use a spreadsheet-type annotation input system (Figure 1B). The sheet looks like a table having rows whose number is equal to number of nucleotide sequences and columns including source feature and a preset feature (e.g., source + CDS, source + rRNA). Though, submitter cannot add, delete and change the preset feature keys, submitter can easily know required feature and qualifier keys for the annotation because required qualifiers are preselected from the start (see (iii) and (iv) below). Useful functions, such as copying a value downward or pasting text list to multiple entries, are available in a spreadsheet-type annotation input system, helping the submitter to shorten the time necessary to complete the submission.
- Specialized templates have been prepared for data submission, including bacterial 16S rRNA, mRNA, influenza A virus gene, D-loop, etc. Each template has feature keys that must be used in the annotation.
- Available qualifier keys are automatically chosen with each template. For example, if the submitter selects a template from ‘Sequences from isolated bacteria or archaea’, qualifiers used in source feature are limited to those relevant to bacteria or archaea.
- In the system, qualifier keys are classified into three ranks: ‘mandatory’, ‘recommended’ and ‘optional’, according to the priority of usage. The ‘mandatory’ and ‘recommended’ qualifiers are automatically displayed on the screen to inform the submitter what information is required for the annotation. The ‘optional’ qualifiers are manually added by the submitter if needed.
- The new submission system can receive multi-FASTA format, which means that a user can submit multiple nucleotide sequences at once.

D-easy is fast and requires low effort to prepare a submission of multiple nucleotide sequences. It is available from http://www.ddbj.nig.ac.jp/sub/websub-e.html.

### Sequence analytical services: WebBlast, ClustalW and DRA Pipeline

The Web BLAST (13) services of DDBJ are hosted on the supercomputer system of the NIG (1). The NIG supercomputer system (whose operation started in March 2012) is a typical HPC cluster system, consisting of general-purpose calculation nodes (thin-node 64 GB memory, 352 nodes) and calculation nodes for memory-intensive tasks including *de novo* assembly of NGS data (2 medium nodes each with 2 TB of memory and 1 fat node with 10 TB of memory). These calculation nodes are interconnected with InfiniBand QDR by a complete bisection fat-tree topology. In addition, to allow the many calculation nodes to read and write the same files in parallel, the NIG supercomputer is equipped with 2 PB of the Lustre parallel distributed file system (http://www.lustre.org/) as a high-performance large external storage system.

Web BLAST service of DDBJ is hosted on several Web servers in the NIG supercomputer system and receives user requests (query sequences) from a Web input form or a file upload form. DDBJ also provides the newer version of Web API for Bioinformatics (WABI) (14–16), a RESTful Web API service that can process many requests sent from computer programs.

Requests sent both to Web applications and to Web APIs are funneled to a resource scheduler, the Univa Grid Engine (UGE, http://www.univa.com/products/grid-engine.php), on the NIG supercomputer system. UGE manages the requested jobs in the work queue and allocates calculation nodes to each job. This configuration drastically decreases the workload of the Web servers so that they can accept as many as 1000 requests per second. This ability is important for large-scale data analysis using Web APIs. At present, 28 thin nodes (448 cores) of the NIG supercomputer are assigned for the Web BLAST and WABI services. By the beginning of March 2014, the NIG supercomputer system will be enhanced to accommodate the increasing demands of massive data analysis. This enhanced system will be equipped with 7 PB of external storage in the Lustre system and ∼550 general-purpose calculation nodes. Along with enhancement, the performance of the Web applications and Web APIs will be improved to a considerable extent.

Other Web applications and RESTful Web APIs working in similar ways are planned for release. The VecScreen system (http://www.ncbi.nlm.nih.gov/tools/vecscreen/univec/), ClustalW (17,18), MAFFT (19,20) and a keyword search system for DDBJ flat files will be released in the near future. Our current keyword search system, ARSA (1), searches against only a single database, traditional DDBJ flat files. It is expected that target database of search system will be expanded to all DDBJ databases because API-based search indexing is also applicable to other databases, such as DRA, BioProject, BioSample, etc.

The DDBJ Read Annotation Pipeline (DDBJ Pipeline, http://p.ddbj.nig.ac.jp/) is a high-throughput Web annotation system of NGS reads running on the NIG supercomputer (21). The DDBJ Pipeline offers a user-friendly graphical Web interface and processes massive NGS datasets using decentralized processing by NIG supercomputers, which is currently free of charge. The pipeline consists of two analysis components: basic analysis for reference genome mapping and *de novo* assembly and subsequent high-level analysis of structural and functional annotations. The high-level analysis employs a non-original Galaxy interface (22), given that the diverse workflows require flexible connections with respective analytical programs. Public NGS reads from the DRA located on the same supercomputer can be imported into the pipeline via input of only an accession number. In the 2013 update, the functional enhancement of DDBJ Pipeline covers new three workflows: *de novo* transcriptome annotation using trinity (23), DNA polymorphism detection with multiple strains, and human HLA haplotype detection (24). As an extension of the DDBJ Pipeline, we are now preparing to develop a virtual machine image.

## FUTURE DIRECTION

In this report, we have introduced the 2013 update of DDBJ including a thorough renovation of the submission system D-easy for the INSDC conventional nucleotide database. At present, D-easy works independently and is not connected to other DDBJ database services. We are planning to connect D-easy under the D-way system to enable users to submit their comprehensive data to relevant DDBJ databases such as the traditional DDBJ, DRA, BioProject and BioSample. We are also improving the organism input and template systems to increase the variety of templates involving many annotation types.

DDBJ is promoting the integration of databases using Semantic Web technology in cooperation with the Database Center for Life Science (DBCLS, http://dbcls.rois.ac.jp/en/) and NBDC. We are developing the INSDC/DDBJ ontology as a standardized, systematic description of a DDBJ sequence entry, including information about submitters, references, source organisms and biological feature annotations. A Web Ontology Language for INSDC Feature Table Definition, which is a common annotation document revised by INSDC once a year, has been generated (25). In the DDBJ nucleotide submission system, we have planned for the use of SPARQL querying of the INSDC/DDBJ ontology as the submission system configuration instead of just updating the submission system each year. In the near future, we will support the RDFization of DDBJ nucleotide sequence entry and a public SPARQL endpoint toward the integration of DDBJ resources including the submission system and public release of data.

## ADDITIONAL INFORMATION

More information is available on the DDBJ website at http://www.ddbj.nig.ac.jp. News is delivered by really simple syndication (RSS), Twitter and mail magazines. Instructional videos of PowerPoint slides for explaining the DDBJ submission system are also available through ‘DDBJ YouTube Channel’ on YouTube.

## FUNDING

Ministry of Education, Culture, Sports, Science and Technology of Japan (MEXT) via a management expense grant for Inter-University Research Institute Corporation to the DDBJ; Grant-in-Aid for Scientific Research on Innovative Areas (Genome Science) to DDBJ, SRA and DDBJ Pipeline (partial). Funding for open access charge: MEXT management expense grant to the DDBJ.

*Conflict of interest statement.* None declared.
